# Molecular Epidemiology of GI.3 Norovirus Outbreaks from Acute Gastroenteritis Surveillance System in Taiwan, 2015–2019

**DOI:** 10.1155/2020/4707538

**Published:** 2020-02-12

**Authors:** Shu-Chun Chiu, Jia-Kai Hsu, Szu-Chieh Hu, Ching-Yi Wu, Ying-Chin Wang, Jih-Hui Lin

**Affiliations:** ^1^Center of Diagnostics and Vaccine Development, Centers for Disease Control, Taipei 11561, Taiwan; ^2^Department of Bioscience and Biotechnology, National Taiwan Ocean University, Keelung 20224, Taiwan

## Abstract

Norovirus is the leading cause of food-borne disease outbreaks. We conducted this study to examine the incidence and molecular characteristics of norovirus genogroup I infections from acute gastroenteritis outbreaks in Taiwan. Between January 2015 and June 2019, 2121 acute gastroenteritis clusters were reported to Taiwan CDC, of which 351 (16.5%) clusters were positive for NoV GI, and GI.3 was the most prevalent (36.8%) during the study period. The GI.3 infections were significantly higher than non-GI.3 infections in the age groups of 0–5 and 6–18 years. The phylogenetic analysis of the MCC tree revealed that VP1 genes were divided into 3 groups: the GI.P3-GI.3 strains in Taiwan were genetically close to Japan and the GI.Pd-GI.3 strains were segregated into 2 other groups which were genetically closely related to China. In addition, 7 GI.Pd-GI.3 recombinants were identified circulating in Taiwan between 2018 and 2019, and the prevalence of GI.Pd-GI.3 should be monitored to assess whether this could become the new predominant strains in neighboring Asian countries or other parts of the world. Both GI.P3-GI.3 and GI.Pd-GI.3 strains cocirculate, the recombination among these two lineages occurs frequently, contributing to the genetic diversity and multiple occurrences of different norovirus lineages, and their rapid evolution makes future control more difficult. Continued surveillance and timely interventions are critical to understand the complexity of norovirus gene variation and to monitor the new emerging norovirus strains.

## 1. Introduction

Human noroviruses (NoVs) are the main causative agent of nonbacterial acute gastroenteritis [1]. They are a genetically diverse group of single-stranded positive-sense RNA viruses with 7.7-kb genomes divided into three open reading frames (ORFs). ORF1 encodes a large polyprotein that is cleaved into six mature nonstructural proteins, ORF2 encodes a major structural protein called VP1 that can self-assemble into virus-like particles (VLPs), and ORF3 encodes a minor structural protein called VP2 [2, 3]. NoVs are highly diverse viruses that can be genetically grouped into 10 genogroups (GI–GX), but only genogroups GI, GII, GIV, GVIII, and GIX can infect humans, with the GII genogroup being the most prevalent [4, 5]. Each genogroup can be further classified into numerous genotypes based on the sequence differences of their VP1 proteins. To date, 9, 22, and 2 VP1 genotypes have been recognized in GI, GII, and GIV, respectively [5, 6]. As recombination frequently occurs in the ORF1/ORF2 overlap, genotyping of both RNA-dependent RNA polymerase (RdRp) in ORF1 and VP1 in ORF2 is necessary to establish a recombinant identity to the virus [7, 8].

In Taiwan, human noroviruses are the common cause of acute gastroenteritis (AGE) outbreaks and are the major cause of both all-age-group diarrhea and food-borne disease outbreaks [9–11]. According to the Communicable Disease Control Act, all suspected gastroenteritis outbreaks must be reported and stool samples must be collected to Taiwan CDC through the Notifiable Diseases Surveillance System. This surveillance system aimed to control the spread of infectious diseases including NoV infections by monitoring the circulating strains. Laboratory-confirmed NoV GI.3 significantly increased in 2018, implying a wave of epidemic of GI.3 occurred in Taiwan. Here, we presented the epidemic strains of norovirus GI.3 with the P2-domain mutation and the recombinant norovirus GI.Pd-GI.3 strains circulating in Taiwan between 2015 and 2019, and to characterize their epidemiological aspects, in particular the link between molecular epidemiologic and phylogenetic characterization.

## 2. Materials and Methods

### 2.1. Sample Collection

Outbreaks were defined as including two or more cases of gastroenteritis linked in place and time. A new outbreak was arbitrarily defined as occurring at least 7 days after the last case in a previous outbreak or as occurring in a different patient care unit such as a ward or hospital [12, 13]. Stool samples from acute gastroenteritis outbreaks were collected from January 2015 to June 2019 in Taiwan. Acute gastroenteritis cases were defined as vomiting or diarrhea (have three or more loose or liquid stools per day). The biological materials in this study were used for standard diagnostic procedures following the physician's prescriptions and were conducted in accordance with no specific sampling and no modification of the sampling protocol. Following local regulations, the procedure did not require specific consent from patients.

### 2.2. Detection of Norovirus

Specimens from patients were submitted to Taiwan CDC for bacterial and viral tests. Bacterial examinations included cultures for common enteric bacteria, such as *Salmonella*, *Shigella*, *Vibrio cholerae*, *Vibrio parahaemolyticus*, pathogenic *E. coli*, *Staphylococcus aureus*, and *Bacillus cereus*, while viral tests included real-time reverse transcription polymerase chain reaction (rRT-PCR) for norovirus and rotavirus as previously described [14–16]. All norovirus-positive samples in rRT-PCR were PCR amplified at the ORF1/ORF2 junction and were sequenced with primers as previously described [17]. Genotypes were assigned using online Norovirus Genotyping Tool Version 2.0 [18] available at https://www.rivm.nl/mpf/typingtool/norovirus/ and the Human Calicivirus Typing Tool [5] available at https://norovirus.ng.philab.cdc.gov.

### 2.3. Sequence Analysis

The partial sequences of the RdRp gene and nearly full-length (1599 bp) coding sequences of VP1 were amplified by RT-PCR using PrimeScript One Step RT-PCR Kit (Takara Bio, Inc., Japan) and sequenced with the primers as previously described [17, 19, 20]. Sequences obtained in this study have been deposited in GenBank and assigned accession numbers MN922648–MN922742.

### 2.4. Phylogenetic Characterization

Pairwise alignment was performed using BioEdit 7.2.5, while multiple sequence alignment was performed using MUSCLE 3.8 [21], where the aligned sequences were further manually inspected and edited. Phylogeny reconstruction and evaluation were implemented in BEAST 1.10.1 using the Bayesian Markov chain Monte Carlo (MCMC) [22] method. In brief, jModelTest2 [23] is used to select the best-fit nucleotide substitution model and then to determine the appropriate clock and tree model by path sampling/stepping-stone sampling (PS/SS) in BEAST 1.10.1. Maximum clade credibility (MCC) trees were then constructed using the TreeAnnotator program in BEAST [24] and visualized using FigTree 1.4.4 (https://github.com/rambaut/figtree/releases). Potential recombinant sequences were detected, and the localization of possible recombination break points was determined using Recombination Detection Program v.4.16 (RDP4) [25]. A multiple-comparison-corrected *p* value cutoff of 0.05 was used throughout. The recombination events were further confirmed along with breakpoints using the SimPlot program [26]. SimPlot analysis was performed by setting the window width and the step size to 200 bp and 20 bp, respectively. Selection pressure analysis was performed using the software available in the Datamonkey software package (http://datamonkey.org) [27]. The genomic sequences used as reference sequences were retrieved from the National Center for Biotechnology Information (NCBI).

### 2.5. Statistical Analysis

Categorical variables were analyzed by chi-square and Fisher's exact tests [28]. Odds ratio (OR) and 95% confidence interval were calculated by binary logistic regression. All statistical tests were two-sided, and *p* value less than 0.05 was considered statistically significant.

### 2.6. Ethical Approval

This study was approved by the Institution Review Board of Taiwan Centers for Disease Control (No. IRB108102). The consent was waived for this study as there was no personal information collected from subjects.

## 3. Results

**Figure 1:**
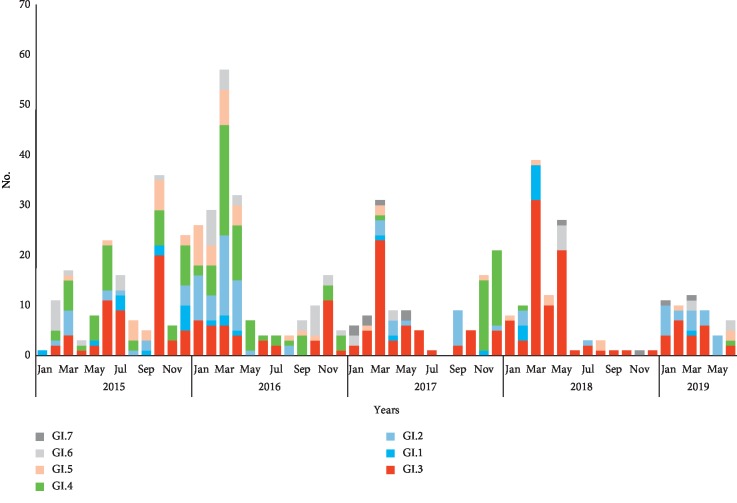
Seasonal distribution of NoV GI genotypes in Taiwan, 2015–2019.

Surveillance of clusters of AGE in Taiwan is based on the reporting system from schools, populous institutes, and laboratory diagnostics. A total of 2121 AGE clusters were reported to Taiwan CDC during the study period of 2015 to 2019. Of them, 351 (16.5%) clusters were positive for NoV GI, and GI.3 was the most frequently detected genotype (36.8%), followed by GI.4 (21.7%), GI.2 (18.5%), and the other GI genotypes (23%, including GI.1, GI.5, GI.6, and GI.7). Although the relative prevalence of NoV GI varies from season to season, GI.3 was the major epidemic strain during the study period, the percentage of GI.3-positive clusters increased from 2017 to 2019, and the viral detection rate was 43.7% in 2017, 66.7% in 2018, and 39.5% in 2019 (counted to July). This reflects GI.3 was the predominant NoV GI genotype circulating in patients with acute diarrhea in Taiwan and with a peak in February to April (Figure 1). A total of 760 cases from 351 clusters were laboratory-confirmed NoV GI positive, 261 (27.6%) of them were GI.3 positive with age ranging from 10 months to 89 years, and 118 (45.2%) of these GI.3-positive cases are children and teenagers (less than 18 years old). A binary regression logistic analysis was performed to determine significant associations, using 19- to 30-year groups which had the robust immune system as the reference group, and the GI.3 infections were significantly higher than non-GI.3 infections in the age groups of 0–5 and 6–18 years (OR = 2.25, *p*=0.0229; OR = 1.53, *p*=0.0461). However, there is no statistical difference between the elder group and the reference group. The detection rate between males and females was not significantly different in each age group (Table 1).

**Figure 2:**
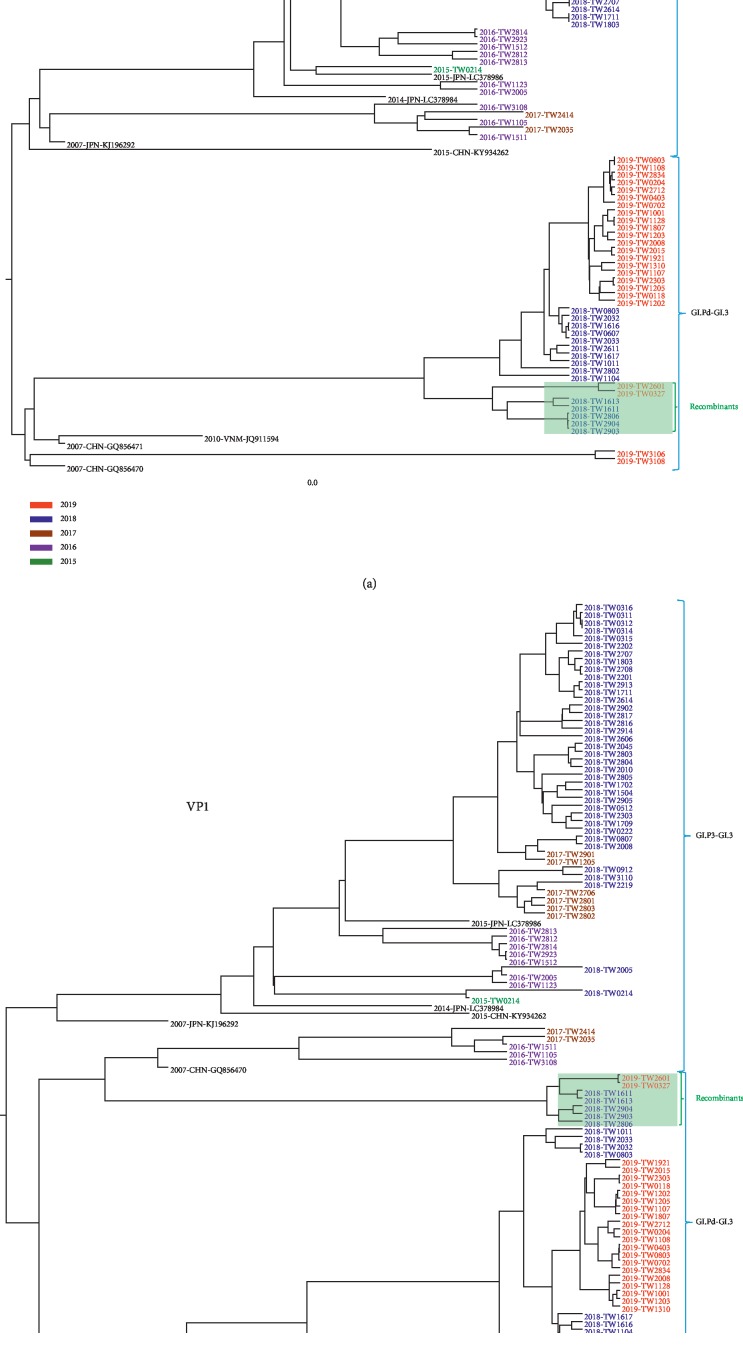
Phylogenetic analysis of Taiwan GI.3 RdRp genes (a) and VP1 genes (b) during 2015–2019. The RdRp tree was constructed from 708 bp (nucleotide positions 4694–5401 based on the Hu/GI/Otofuke/1979/JP reference strain) (accession number AB187514), and the VP1 tree was constructed from 1599 bp (nucleotide positions 5388–6986 based on the reference strain AB187514). The phylogeny of time-scaled analysis was summarized from MCMC phylogenies of the RdRp and VP1 genes by using a relax-clock model with uncorrelated lognormal distribution in BEAST.

**Figure 3:**
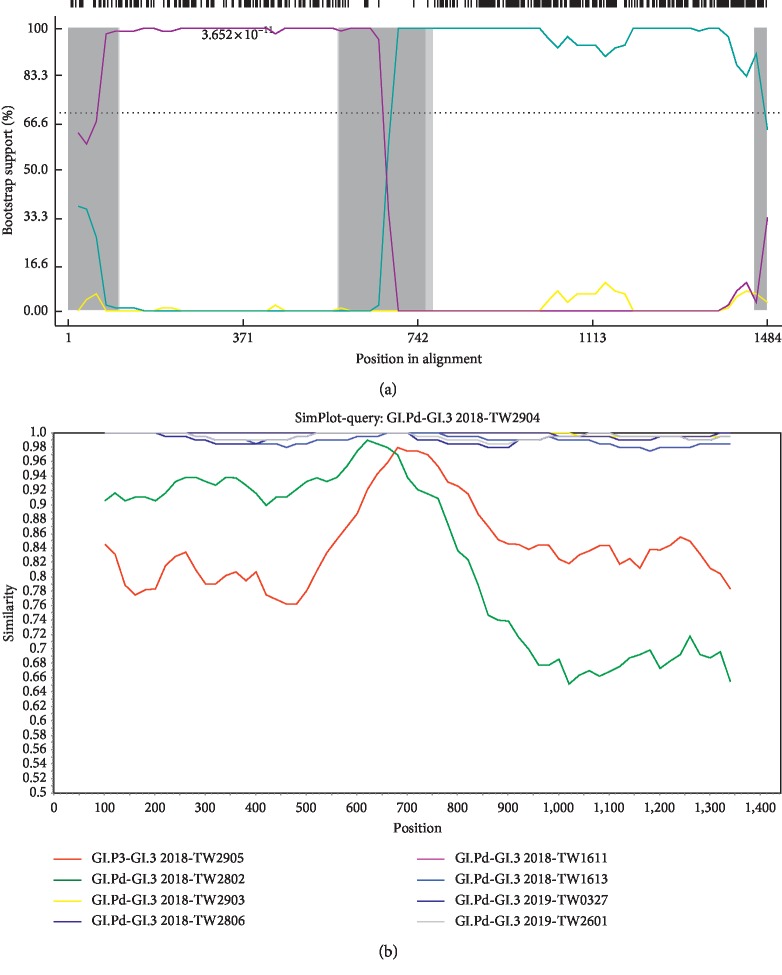
Recombination analysis of Taiwan GI.3 norovirus. (a) Bootscan analysis of recombinant GI.3 norovirus using RDP software version 4.0. Bootscan evidence for the recombination event is based on pairwise distance. (b) SimPlot analysis using the recombinant GI.Pd-GI.3 virus (GI.Pd-GI.3 2018-TW2904) as the query sequence and making use of a sliding window of 200 bp with a step size of 20 bp. The Kimura 2-parameter model is applied. The *y*-axis shows the percentage of similarity between the selected sequences and the query sequence.

The nearly full-length PCR amplicon (1599 bp) of the VP1 gene and partial RdRp gene nucleotide sequences were randomly selected by different outbreaks and months. A total of 95 GI.3 strains determined in this study further generated Bayesian phylogenetic trees for time-scaled analysis in Figures 2(a) and 2(b) for the RdRp gene and VP1 gene, respectively. The MCC tree showed that the VP1 genes and RdRp genes were divided into 3 groups: the GI.P3-GI.3 strains found in Taiwan were genetically close to Japan and the GI.Pd-GI.3 strains were segregated into 2 other groups which were genetically closely related to China (Figure 2). In addition, most of GI.3 RdRp genes broadly fall into 2 groups (GI.P3-GI.3 and GI.Pd-GI.3), whereas 7 GI.Pd-GI.3 strains were found to be the recombinant strains, the genotyping analyses of which by phylogenetic trees of RdRp and VP1 genes showed discordance of GI.3 genogroups (Figure 3).

Among these 7 recombinant strains, nucleotide identity ranged from 98.9% to 99.8%. The recombination breakpoints observed in 7 recombinant strains detected in the present study were located at nucleotide position 618 in PCR amplicons in this study (2293 bp), corresponding to nucleotide position 5346 in relation to the Hu/GI/Otofuke/1979/JP reference strain (accession number AB187514), localized in the ORF1/2 junction. The recombination point determined by both RDP4 and SimPlot programs showed similar results (Figures 3(a) and 3(b)). SimPlot analysis was performed using the recombinant GI.Pd-GI.3 virus (GI.Pd-GI.3 2018-TW2904) as a query sequence. Data showed that GI.P3-GI.3 2018-TW2905 and GI.Pd-GI.3 2018-2802 are the parent sequences, and the other 6 GI.Pd-GI.3 strains (2018-TW2903, 2018-TW2806, 2018-TW1611, 2018-TW1613, 2019-TW0327, and 2019-TW2601) are highly similar to the query recombinant strain 2018-TW2904 (Figure 3(b)).

To estimate comprehensively the positive selection site in the VP1 protein of NoV, single likelihood ancestor counting (SLAC), fixed-effects likelihood (FEL), internal fixed effects likelihood (IFEL), and mixed-effects model of evolution (MEME) were applied (Table 2). The method implemented in Datamonkey detected 2 sites (377 and 505) from both FEL and MEME as potentially episodic positive selection and suggested these sites may play an important role during the adaptive evolution of the GI.3 strain to local environments. Furthermore, the evolutionary rate of the VP1 gene was estimated to be 1.903 × 10^−3^ substitutions/site/year (95% HPD interval, 1.461–2.343 × 10^−3^ substitutions/site/year), and the overall RdRp gene region of Taiwan GI.3 strains was estimated to be 2.576 × 10^−3^ substitutions/site/year (95% HPD interval, 1.878–3.264 × 10^−3^).

## 4. Discussion

Surveillance of viral enteric diarrhea in Taiwan is performed by sentinel physicians through the Notifiable Diseases Surveillance System and is based on laboratory detection of virus nucleic acid. This study represents the prevalence, epidemic genotypic diversity, and spatiotemporal dynamics of NoV GI.3 genotype strains in Taiwan from 2015 to 2019. From our data, it is seen that the prevalence of NoV GI in Taiwan is higher than previous reports in China, Seoul, and Thailand [29–31], NoV GI.3 was the most common genotype detected in outbreaks of NoV GI among Taiwanese people, and NoV GI.3 infection mainly occurred in preschool students (0–5 years) and school students (6–18 years) in Taiwan, similar to previous NoV reports from China that NoV outbreaks mainly occurred in kindergartens and primary schools [29, 32]. However, it is worth noting that our data reveal NoV outbreaks in teenager school students, the risk factors for norovirus infection and the origin of these school outbreaks are not clear, but the epidemiological surveillance data show that norovirus infections increase when school is in session especially during the beginning of the term and decrease in summer and winter vacation. This inconsistent result might be due to different school cultures from other countries; for example, school group meal service is common in Taiwan.

Phylogenetic analysis showed the RdRp region of NoV GI.3 strains formed two distinct clusters: GI.P3-GI.3 strains were observed in 2015–2019, whereas the GI.Pd-GI.3 strains including recombinants were only detected in 2018 and 2019. Then, different variants of GI.3 norovirus cocirculate simultaneously in Taiwan, and even in a city, the virulence and transmissibility of GI.3 strains were likely enhanced through mutation, contributing to its recent dissemination together with different subgenotypes. RdRp of norovirus is a key enzyme responsible for viral transcription and replication [33] and was suggested to be a driving factor in norovirus recombination [34]. A previous study showed that the low prevalence of norovirus is a consequence of a low mutation rate in RdRp, resulting in limited antigenic drift and an inability to escape herd immunity compared to the predominant strains; the suggested mutation rate in combination with a high replication rate is a key determinant in epidemiological fitness [35].

Genetic recombination is a common phenomenon in norovirus, which has a major impact on its evolution and genotype diversity. As most NoV recombinant occurs in a single hotspot breakpoint located in the ORF1/2 overlapping region [25, 34], a combined characterization of both the polymerase and VP1 regions is important to monitor new NoV genotype emergence and recombinant strains. In this study, 7 GI.Pd-GI.3 recombinants were identified circulating in Taiwan between 2018 and 2019, and this result is inconsistent with other studies showing that less GI norovirus recombinants have been described when compared to GII norovirus recombinants [1, 33, 36]. The prevalence of GI.Pd-GI.3 should be monitored to assess whether this could become the new predominant strains in neighboring Asian countries or other parts of the world. Furthermore, spatial reconstruction of the VP1 gene of the GI.3 genotype indicated that the 3 groups of GI.3 viruses found in Taiwan were genetically closely related to Japan and China, and this might be due to traveling frequently for business and sightseeing among Taiwan, Japan, and China. In addition, from our time-scaled phylogenetic analysis, it is seen that the GI.3 genotype accelerated in variation and showed transmission dynamics, and variants in 2019 are not identical to its parent strain in 2018, as evidenced in the phylogenetic tree; despite their evolution from Japan or China, 2018 formed at least 3 distinct groups.

The results presented in this study demonstrate that genetically distinct viruses within both GI.P3-GI.3 and GI.Pd-GI.3 strains cocirculate and that recombination between these two lineages occurs frequently, contributing to the genetic diversity of the circulating strains. Within the 5-year surveillance, the GI.3 strain accumulated nearly 23.2% (76.7%–99.9%) genetic distance; multiple occurrences of different norovirus lineages and their rapid evolution make future control more difficult because prior exposure to certain norovirus variants cannot offer complete protection from new variant infection. Continued surveillance and unified systems for norovirus typing are critical to monitor the emergence and impact of these GI.3 strains and other new norovirus strains.

## Figures and Tables

**Table 1:** Association between age and infection of the norovirus GI genotype in Taiwan, 2015–2019.

Age group	GI.3 (*N* = 261)	Other GI genotypes (*N* = 499)	Odds ratio^*∗*^	*p* value	GI.3 (*N* = 261)		Other GI genotypes (*N* = 499)	
	*n* (%)	*n* (%)			Male, *n* (%)	Female, *n* (%)	Male, *n* (%)	Female, *n* (%)
0–5 yrs	19 (7.3)	22 (4.4)	2.25	0.0229	13 (68.4)	6 (31.6)	13 (59.1)	9 (40.9)
6–18 yrs	99 (37.9)	169 (33.9)	1.53	0.0461	58 (58.6)	41 (41.4)	101 (59.8)	68 (40.2)
19–30 yrs	48 (18.4)	125 (25.1)	1.00	Reference	34 (70.8)	14 (29.2)	83 (66.4)	42 (33.6)
31–45 yrs	50 (19.2)	87 (17.4)	1.50	0.1007	26 (52.0)	24 (48.0)	46 (52.9)	41 (47.1)
46–65 yrs	40 (15.3)	77 (15.4)	1.35	0.2424	14 (35.0)	26 (65.0)	32 (41.6)	45 (58.4)
>65 yrs	5 (1.9)	19 (3.8)	0.69	0.4763	2 (40.0)	3 (60.0)	4 (21.1)	15 (78.9)

^*∗*^Calculated by logistic regression. The total number of clusters and cases of NoV GI infection is 351 and 760, respectively.

**Table 2:** Positive selection analysis using SLAC, FEL, and MEME methods.

Positive selection sites					
Data set	Mean d*n*/d*s*	SLAC^a^ (*p* value)	FEL^b^ (*p* value)	MEME^c^ (*p* value)	
VP1	0.056		377 (0.055)	20 (0.03)	302 (0.03)
			505 (0.084)	377 (0.07)	505 (0.00)
				508 (0.07)	528 (0.00)
				532 (0.04)	
RdRp	0.035	82 (0.09)		43 (0.03)	111 (0.01)

^a^SLAC: single likelihood ancestor counting: codons with *p* value < 0.1. ^b^FEL: fixed-effects likelihood: codons with *p* value < 0.1. ^c^MEME: mixed-effects model of evolution: codons with *p* value < 0.1.

## Data Availability

The datasets used and analyzed during the current study are available from the corresponding author on reasonable request.
